# The effect of femtosecond laser-assisted *in situ* keratomileusis on contrast sensitivity

**DOI:** 10.3389/fnins.2024.1326572

**Published:** 2024-08-29

**Authors:** Pinqing Yue, Xiang Wang, Zeng Wang, Ying Li, Di Wu, Hua Zhang, Pan Zhang

**Affiliations:** ^1^Department of Psychology, Hebei Normal University, Shijiazhuang, China; ^2^College of Career Technology, Hebei Normal University, Shijiazhuang, China; ^3^Department of Psychology, Hebei Medical University, Shijiazhuang, China; ^4^Department of Psychiatry, Beijing Children’s Hospital, Capital Medical University, National Center for Children’s Health, Beijing, China; ^5^Department of Medical Psychology, Air Force Medical University, Xi’an, China; ^6^Department of Ophthalmology, Shijiazhuang People’s Hospital, Shijiazhuang, China

**Keywords:** femtosecond laser-assisted *in situ* keratomileusis, contrast sensitivity, spatial frequency, external noise, perceptual template

## Abstract

The benefits of femtosecond laser-assisted *in situ* keratomileusis (FS-LASIK) for correcting vision, particularly in terms of spherical equivalent (SE) and visual acuity (VA), have gained broad recognition. Nevertheless, it has remained uncertain whether FS-LASIK has a positive impact on contrast sensitivity (CS). In this study, we measured CS on seven participants by the quick contrast sensitivity function (qCSF) and compared CS before and after the surgery at two time points (1 day and 7 days after) by the repeated measures analysis of variance (ANOVA). Then, we clarified the underlying mechanisms using the perceptual template model (PTM). Furthermore, we investigated the relationship among SE, VA, and CS employing the Pearson correlation test. We found that (1) CS exhibited significant improvements on postoperative day 1, with further enhancements observed up to postoperative day 7, (2) CS improvements were dependent on spatial frequency (SF) and external noise, (3) CS improvements were attributed to the reduction of internal noise and the enhancement of the perceptual template, (4) VA and SE demonstrated significant improvement post-surgery, and (5) no significant correlations were observed among SE, VA, and CS, possibly due to limitations in sample size and lighting conditions. These findings contribute to our comprehension of FS-LASIK and provide a great indicator for assessing the outcomes of visual surgery.

## Introduction

Femtosecond laser-assisted *in situ* keratomileusis (FS-LASIK) is a widely utilized surgery aimed at correcting myopia. This surgical method is recognized for its safety, effectiveness, and predictability (Biscevic et al., 2020; Lim et al., 2020; Zhang et al., 2020; Du et al., 2023). One of its main advantages is the shorter visual recovery time compared to other procedures such as photorefractive keratectomy (PRK) and small incision lenticule extraction (SMILE) (Bashir et al., 2017; Chang et al., 2022). In addition to improving vision, FS-LASIK can also enhance the quality of life and mental health (Han et al., 2020). For example, patients who undergo this surgery often experience reduced eye fatigue and increased self-esteem following treatment (Klokova et al., 2019).

Most studies on FS-LASIK evaluate surgical quality by measuring visual acuity (VA) and spherical equivalent refraction (SE), and it is crucial to consider contrast sensitivity (CS) as well. CS represents the threshold contrast for seeing a target under a range of spatial frequency (SF), whereas VA is detected at high contrast (at least 85%) and limited SFs (Owsley, 2003; Zimmerman et al., 2011). Notably, certain studies have uncovered cases in which patients with conditions such as cataracts and diabetes exhibited impaired CS despite having normal VA (Pramanik et al., 2020; Xiong et al., 2020), underscoring the sensitive nature of CS as an indicator of visual performance. Despite some studies focusing on changes in CS before and after FS-LASIK, their findings have shown inconsistency due to various factors.

In previous investigations, various measurement methods have been employed. Some studies applied conventional CS tests, such as the CVS-1000E test (Liu et al., 2016; Hashemi et al., 2017) with pre-determined frequencies and contrast levels, consequently yielding restricted data insights. In contrast, other research endeavors embraced a new method called the quick contrast sensitivity function (qCSF). This method gathers data across all SFs, resulting in a complete CSF derived from a mere 50 trials. It demonstrates great accuracy and precision (Lesmes, 2010; Hou et al., 2016) and should be used in the field of FS-LASIK. Although some researchers have evaluated FS-LASIK using qCSF, the modulation of CS improvement by SF remains unknown. For example, Gao et al. (2022) measured CS from 1.5 to 18 cpd by the qCSF and compared the total CS values before and after FS-LASIK, neglecting to analyze CS change at each SF. Consequently, it remains uncertain whether the improvements in postoperative CS depend on SF. In this study, we used the qCSF to assess the effect of FS-LASIK on CS across 10 SFs.

Moreover, external noise is another important factor affecting CSF that has been ignored in previous studies. For example, drivers’ visual performance can be compromised under adverse conditions such as driving in inclement weather or on uneven road surfaces (Horswill and Plooy, 2008; Spreng et al., 2018). Georgeson and Sullivan (1975) have demonstrated that the flattening of the CSF can be observed when a high density of external noise is added to a contrast detection task. In addition to simulating the real world, the equivalent input noise method can help explain the underlying mechanism responsible for CS improvements following FS-LASIK in accordance with the perceptual template model (PTM). The PTM decomposes CS into three intrinsic limitations of the perceptual system (Lu and Dosher, 1999): (1) internal additive noise, which is equal to amplify both signal and noise from input stimuli; (2) internal multiplicative noise, which is related with Weber’s law behavior of the perceptual system; (3) the perceptual template, which helps exclude external noise. PTM has accurately revealed alcohol-induced CS loss and visual perceptual learning (Lu et al., 2011; Zhang et al., 2022).

In summary, the primary objective of this research is (1) to determine how FS-LASIK affects VA, SE, and CS; (2) to assess whether SF and external noise modulate CSF; (3) to investigate the relationship among VA, SE, and CS; (4) to explain the underlying mechanism by the PTM model.

## Methods

### Participants

The G*Power analysis (Faul et al., 2007) conducted for the repeated measures analysis of variance (ANOVA) indicated that a sample size of six participants would be sufficient. This determination was based on a moderate effect size (*f* = 0.25), a desired test power (1 − *β*) of 0.95, and a significance level of *α* = 0.05. Seven participants were recruited and signed informed consent before the surgery, with one eye per participant randomly selected for inclusion in the analysis. The inclusion criteria encompass individuals aged 18 years or older, with refractive stability observed for at least the previous year, a central corneal thickness exceeding 480 μm, and no prior history of ocular surgery or pathology. In accordance with the principles outlined in the Declaration of Helsinki, approval for the study was obtained from the Ethics Committee of Shijiazhuang People’s Hospital and the Ethics Committee of Hebei Normal University.

### Apparatus

Stimuli were generated using MATLAB with PsychToolbox and presented on a luminance-calibrated Apple (CRT) monitor. The monitor had a background brightness of 36.3 cd/m^2^, a resolution of 1,280 × 1,024, and a refresh rate of 85 Hz. Subjects sat 1.76 m away from the monitor. Flaps were created using a 500 kHz VisuMax femtosecond laser (Carl Zeiss Meditec AG) during the FS-LASIK procedure. The diameter of the flaps was set to 8.5 mm, and the flap thickness was 100 μm. The hinges were set at 90°, with a hinge length of 3.53 mm. Stromal tissue ablation was performed using a MEL-90 excimer laser (Carl Zeiss Meditec AG) with a repetition rate of 500 kHz. The optical zone was set at 6–6.5 mm, with a 2 mm transition zone.

### Stimuli

There were vertical gratings with constant cycle (*N* = 3) at 10 SFs (0.5, 0.67, 1, 1.33, 2, 2.67, 4, 5.33, 8, and 16 cpd) under three external noise conditions [*μ* = 0 and *σ* ∈ (0, 0.12, and 0.24)]. The size of the gratings exhibited an inverse relationship with their SF. To blur the edges, the gratings were enveloped by truncated Gaussian masks. The masks were generated using noise images with pixel contrasts following a Gaussian distribution. The sizes of both noise images and gratings were identical. Each noise image contained the same number of gray elements (15 × 15), ensuring that the ratio of spectral energy remained comparable between the noise images and the gratings across all SF values.

### Procedure

VA was assessed using the E-chart at a distance of 5 meters and quantified as the logMAR score for subsequent statistical evaluations. SE was obtained by adding spherical power (DS) and half the cylinder power (DC) together. DS and DC were assessed by a TOPCON RM-8900 autorefractor.

**Figure 1:**
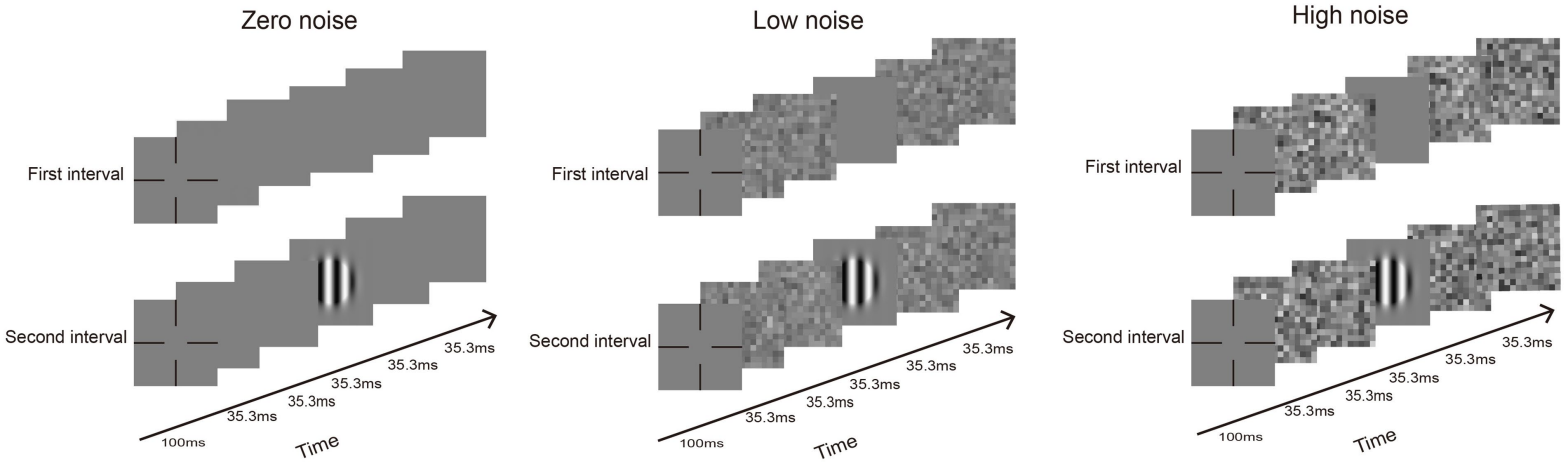
An exemplification of a standard trial conducted across three different noise conditions: absence of noise, low noise, and high noise. A trial is composed of two intervals. In the noise condition, an interval has four noise images and one grating image. When noise is absent, blanks will display the noise images.

CSF was measured using the qCSF method (Figure 1). The whole procedure encompassed 50 trials for each external noise level (zero, low, and high). Each trial was structured with two intervals, separated by a 500 ms blank screen interval. Within each interval, a series of five images (35.3 ms for each) were displayed. The noise images were changed with noise conditions and were presented randomly across the trials. When noise was present, one blank or grating image was temporally masked by two noise images in front and two noise images behind; when noise was absent, the four noise images were replaced by blanks. Participants were required to identify and react to the grating using a gamepad. Thus, a complete CSF test obtained three noise conditions, each of which included 10 SFs with 50 trials. Each participant was tested only one eye.

### Design

The experiment consisted of three stages: pretest, posttest 1 (the first day after FS-LASIK), and posttest 2 (the seventh day after FS-LASIK). VA, SE, and CS were measured at each time point. VA and SE tests were performed in an illuminated room, followed by a 5 min period of dark adaptation and a 15 min CS measurement.

### Statistical analysis

ANOVA and correlation analyses were conducted on SPSS (26.0, Inc., Chicago, IL, United States). CS obtained at posttest 1 and posttest 2 were compared to the initial measurements under varying SF and levels of external noise using repeated-measures ANOVA. Relationships among changes in SE, VA, and CS were evaluated by the Pearson correlation test.

The PTM model was utilized to examine the underlying mechanism. The model proposes three potential reasons for the enhancement in CS: improving signal (i.e., reducing internal additive noise), eliminating external noise (i.e., enhancing the perceptual template), and reducing internal additive noise. To determine the plausible explanation in this study, external noise levels were manipulated. Specifically, when enhanced signal leads to improved CS, contrast thresholds decrease at low noise levels but remain relatively stable at high noise levels due to heightened noise levels and signal Alternatively, if the enhancement in CS results from excluding external noise, contrast thresholds remain unchanged at low noise levels as they depend on internal additive noise, while they decrease at high noise levels due to the exclusion of external noise. Conversely, if the increase in CS is attributed to reducing internal additive noise, contrast thresholds decrease at both low and high noise levels.

In the PTM, we employed the subsequent formulas to assess an individual’s performance (Bejjanki et al., 2014):


(1) $$d^{\prime }=\frac{{\left(\beta c\right)}^{\gamma }}{\sqrt{{\left(N_{ext}\right)}^{2\gamma }+N_{mul}^{2}\left[{\left(\beta c\right)}^{2\gamma }+{\left(N_{ext}\right)}^{2\gamma }\right]+N_{\mathrm{add}}^{2}}}$$

where *d*′ is an index for the perceptual performance; *β* denotes a contrast gain to the signal; and *γ* indicates the nonlinearity of the system. *N*_ext_, *N*_mul_, and *N*_add_ are on behalf of the contrast of external noise, internal multiplicative noise, and internal additive noise, respectively. For a given d’ score, the threshold contrast (*c_τ_*) can be obtained by Equation 2:


(2) $$\begin{array}{l} \log\left(c_{\tau }\right)=\frac{1}{2\gamma }\log\left[\left(1+N_{mul}^{2}\right){\left(N_{ext}\right)}^{2\gamma }+N_{\mathrm{add}}^{2}\right]\\ \;-\frac{1}{2\gamma }\log\left(\frac{1}{d^{\prime 2}}-N_{mul}^{2}\right)-\log\left(\beta \right) \end{array}$$

To simulate CS improvements induced by FS-LASIK, *A*_a_, *A*_m_, and *A*_f_ are utilized to modify *N*_add_, *N*_mul_, and *N*_ext_; and the values of *A*_a_, *A*_m_, and *A*_f_ were set to 1 before LASIK. We would not change *A*_m_ if the slopes of psychometric functions remained unchanged with time (Xu et al., 2006; Lu and Dosher, 2008). Furthermore, the values of *N*_add_ and *β* were influenced by SFs, whereas SFs had no impact on *N*_mul_ and γ.

Based on Equations 1, 2, *c_τ_* can be denoted by Equation 3:


(3) $$\begin{array}{l} \log\left(c_{\tau }\right)=\frac{1}{2\gamma }\log\left[\left(1+A_{m}^{2}N_{mul}^{2}\right){\left(A_{f}N_{ext}\right)}^{2\gamma }+A_{a}^{2}N_{\mathrm{add}}^{2}\right]\\ \;-\frac{1}{2\gamma }\log\left(\frac{1}{d^{\prime 2}}-A_{m}^{2}N_{mul}^{2}\right)-\log\left(\beta \right) \end{array}$$

## Results

### SE

We applied a repeated-measures ANOVA on SE and found that the impact of the time point exhibited a statistically significant main influence on SE [*F*(2, 12) = 149.521, *p* < 0.001, $\eta_{p}^{2}$ = 0.961]. The least significant difference (LSD) analysis indicated that SE at posttest 1 and posttest 2 was better than that at pretest (pretest: −7.41 ± 1.54 D, posttest 1: −0.07 ± 0.47 D, posttest 2: −0.45 ± 0.30 D; all *p* < 0.001); SE at posttest 2 was poorer than that at posttest 1 (*p* = 0.001). Two participants showed slight overcorrection (less than 0.50 D) at posttest 1 but the refraction stabilized at posttest 2; in addition, SE in four participants showed a decrease at posttest 2 relative to it at posttest 1.

### VA

A repeated-measures ANOVA was conducted on VA. The time point has a significant main effect [*F*(2, 12) = 148.067, *p* < 0.001, $\eta_{p}^{2}$ = 0.961]. The LSD test showed that the performance of VA demonstrated improvements in both posttest 1 and posttest 2 compared to the initial pretest evaluation (all *p* < 0.001), indicating the improvements of VA induced by FS-LASIK. Additionally, VA between posttest 1 and posttest 2 was comparable (*p* = 0.659).

### CS

**Figure 2:**
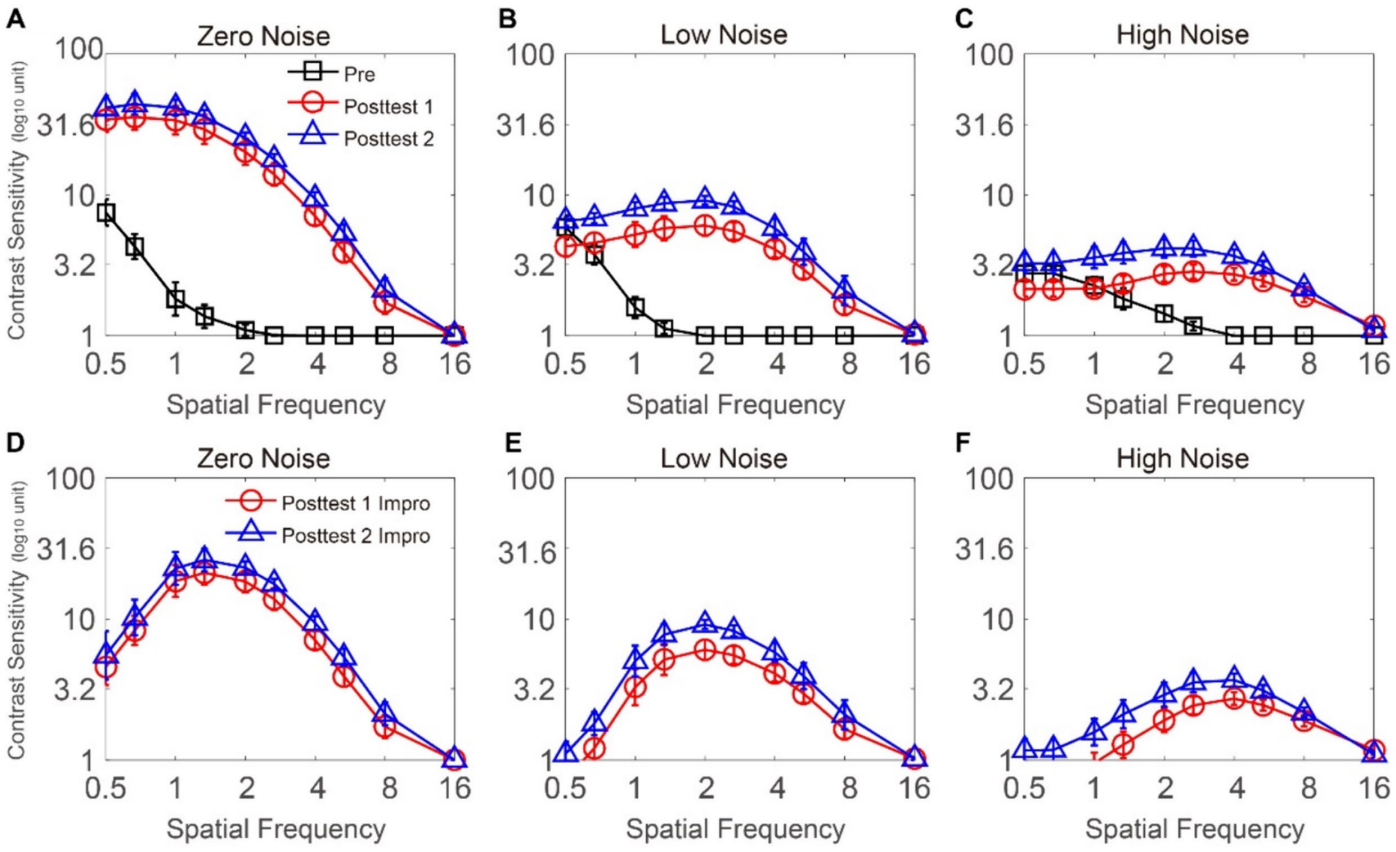
**(A–C)** CSFs under zero-, low-, and high-noise conditions when time points are pretest (dark), posttest 1 (red), and posttest 2 (blue), respectively. **(D–F)** CS improvement functions under zero-, low-, and high-noise conditions when time points are posttest 1 (red) and posttest 2 (blue), respectively. The data collected from each subject were averaged. Standard errors (SEs) were represented by error bars.

The CSF at three noise levels among three time points is plotted in Figures 2A–C. To analyze CS, we employed a repeated-measures ANOVA, considering time point, external noise, and SF as within-subject factors. CS at 16 cpd could not be measured on some subjects at the pretest. To exclude the influence of the floor effect, the data at 16 cpd were excluded. Significant effects were observed for time point, external noise, and SF [*F*(2, 12) = 117.015, *p* < 0.001, $\eta_{p}^{2}$ = 0.951; *F*(2, 12) = 157.552, *p* < 0.001, $\eta_{p}^{2}$ = 0.963; *F*(8, 48) = 113.789, *p* < 0.001, $\eta_{p}^{2}$ = 0.950, respectively]. The two-way interactions were all significant between external noise and time point, between SF and time point, and between SF and external noise [*F*(4, 24) = 48.331, *p* < 0.001, $\eta_{p}^{2}$ = 0.890; *F*(16, 96) = 18.061, *p* < 0.001, $\eta_{p}^{2}$ = 0.751; *F*(16, 96) = 43.5, *p* < 0.001, $\eta_{p}^{2}$ = 0.879, respectively]. Additionally, a significant interaction was observed among the three variables [*F*(32, 192) = 13.52, *p* < 0.001, $\eta_{p}^{2}$ = 0.693]. The LSD test indicated that when external noise was zero, at 0.5–8 cpd, CS at posttest 1 and posttest 2 were significantly greater than that at pretest (all *p* < 0.026) and CS was comparable between posttest 1 and posttest 2 (all *p* > 0.117). The results indicated that, when noise was absent, FS-LASIK improved CS at 0.5–8 cpd. When external noise was low, at 0.5 cpd, CS obtained during the pretest were found to be similar to those recorded during posttest 1 and posttest 2 (*p* = 0.218; *p* = 0.634, respectively). CS at posttest 2 was significantly higher than that at posttest 1 (*p* = 0.019). This was because CS at posttest 1 decreased temporarily relative to that at the pretest and CS at posttest 2 showed slight improvements compared to the pretest; at 0.67 cpd, CS at posttest 2 was the best, while CS at posttest 1 equaled that of the pretest (*p* = 0.02, *p* = 0.023, *p* = 0.416, respectively); at 1–2.67 cpd, CS at posttest 2 was the best, followed by that at posttest 1 and CS at pretest was the worst (all *p* < 0.048); at 4–8 cpd, CS at posttest 1 and posttest 2 significantly increased relative to that at pretest (all *p* < 0.025), and CS showed no significant change from posttest 1 to posttest 2 (all *p* > 0.07). These data revealed that, in the low noise condition, CS became better at 0.67–8 cpd due to FS-LASIK. Specifically, CS at 0.67–2.67 cpd improved up to posttest 1, while CS at 4–8 cpd steadily increased until posttest 2. When external noise was high, at 0.5–1 cpd, CS was comparable among pretest, posttest 1, and posttest 2 (all *p* > 0.076); at 1.33 cpd, CS at posttest 1 was equal to that at pretest and posttest 2 (*p* = 0.292, *p* = 0.062, respectively). CS at posttest 2 was better than that at pretest (*p* = 0.017); at 2–8 cpd, CS at posttest 1 and posttest 2 was higher than that at pretest (all *p* < 0.015) and CS showed no significant change from posttest 1 to posttest 2 (all *p* > 0.087). These analyses indicate that, under high noise conditions, the surgery led to improvements in CS ranging from 1.33 to 8 cpd. CS at 1.33 cpd showed a continuous increase over time, reaching its peak at posttest 2, while CS at 2–8 cpd reached its maximum level at posttest 1. In summary, FS-LASIK could improve on CS at middle-high SF (i.e., 1.33–8 cpd), with the surgical outcome being moderated by external noise levels. Notably, the improvements in CS decreased with increasing noise levels at low SF (i.e., 0.5–1.33 cpd).

In order to investigate the CS improvements in different SF and external noise conditions, we calculated the change at posttest 1 (posttest 1 CS minus that pretest CS) and posttest 2 (posttest 2 CS minus that pretest CS) respectively (as shown in Figures 2D–F). We conducted a repeated-measures ANOVA on CS increase with time point, external noise, and SF as within-subject factors. The data at 16 cpd were excluded due to the floor effect. The main effects of SF and external noise were significant; however, the main effect of time point was not significant [*F*(8, 48) = 26.371, *p* < 0.001, $\eta_{p}^{2}$ = 0.815; *F*(2, 12) = 112.723, *p* < 0.001, $\eta_{p}^{2}$ = 0.949; *F*(1, 6) = 5.897, *p* = 0.051, $\eta_{p}^{2}$ = 0.496, respectively]. Significance was observed exclusively in the interaction between SF and external noise [*F*(16, 96) = 15.907, *p* < 0.001, $\eta_{p}^{2}$ = 0.726]. All other interactions, including the two-way interactions (i.e., time point and external noise, time point and SF), as well as the three-way interaction, were not found to be significant [*F*(2, 12) = 0.573, *p* = 0.578, $\eta_{p}^{2}$ = 0.087; *F*(8, 48) = 0.435, *p* = 0.894, $\eta_{p}^{2}$ = 0.068; *F*(16, 96) = 1.207, *p* = 0.277, $\eta_{p}^{2}$ = 0.167, respectively]. The LSD analysis uncovered a significant distinction between the results of posttest 1 and posttest 2 under each of the noise conditions: in the zero and high noise conditions, there was no obvious difference between posttest 1 and posttest 2 at 0.5–8 cpd (all *p* > 0.117; all *p* > 0.062, respectively); in the low noise condition, CS increase at posttest 2 was better than that at posttest 1 at 0.5–2.67 cpd (all *p* < 0.048); however, the difference between them was not significant at 4–8 cpd (all *p* > 0.07). The LSD test was conducted to compare CS improvements at various SFs between posttest 1 and posttest 2. For CS improvements at posttest 1: (1) when external noise was absent, CS improvements at 1–2.67 was significantly higher than that at 0.5–0.67 cpd and that at 4–8 cpd (all *p* < 0.016; all *p* < 0.016, respectively), except for that at 0.67 and 2.67 cpd, which was comparable (*p* = 0.105); CS increase at 0.5–0.67 cpd had no significant differences with that at 4–8 cpd (all *p* > 0.059), except that CS increase at 0.67 cpd was higher than that at 8 cpd (*p* = 0.005); (2) when noise was low, CS increase at 1.33–4 cpd was significantly better than that at 0.5–1 cpd and that at 5.33–8 cpd (all *p* < 0.019; all *p* < 0.021, respectively), except for those at 1 and 2.67, 1 and 4 cpd, and, 1.33 and 5.33 cpd, which were comparable (*p* = 0.055; *p* = 0.440; *p* = 0.101, respectively). CS increase at 5.33–8 cpd was significantly greater than that at 0.5–0.67 cpd and had no difference with that at 1 cpd (all *p* < 0.039; all *p* > 0.135, respectively), except for 0.67 and 8 cpd, which were comparable (*p* = 0.353). (3) When noise was high, CS increase at 2–8 cpd was better than that at 0.5–1.33 cpd (all *p* < 0.025), except for 1.33 and 8 cpd which was comparable (*p* = 0.191). CS increase at posttest 2 exhibited a similar trend to that observed at posttest 1. For CS increase at posttest 2: (1) in the zero noise condition, CS increase at 1–2.67 cpd was significantly higher than that at 0.5–0.67 cpd and that at 4–8 cpd (all *p* < 0.032; all *p* < 0.05, respectively), except for that at 0.67 and 2.67 cpd, which was similar (*p* = 0.142). CS at 0.5–0.67 cpd had no significant difference with that at 4–8 cpd (all *p* > 0.126), except that CS at 0.67 cpd was better than that at 8 cpd (*p* = 0.015); (2) when low noise was displayed, CS increase at 1.33–4 cpd was significantly greater than that at 0.5–0.1 cpd and that at 5.33–8 cpd (all *p* < 0.049; all *p* < 0.011, respectively) and there was no significant difference between 1 and 2.67 cpd, between 1 and 4 cpd, and between 1.33 and 5.33 cpd (*p* = 0.147; *p* = 0.715; *p* = 0.094, respectively). CS increase at 0.5–1 cpd was comparable with that at 5.33–8 cpd (all *p* > 0.068), except that CS increase at 0.5 cpd was worse than that at 5.33 cpd (*p* = 0.008); (3) when high noise was displayed, CS at 1.33–8 cpd was significantly better than that at 0.5–1 cpd (all *p* < 0.033), except for those at 0.5 and 1.33, and, 1 and 8 cpd, which were comparable (*p* = 0.057; *p* = 0.234, respectively). Together, these results suggest that CS showed the highest improvements at middle SFs under each of the time points and noise conditions.

**Figure 3:**
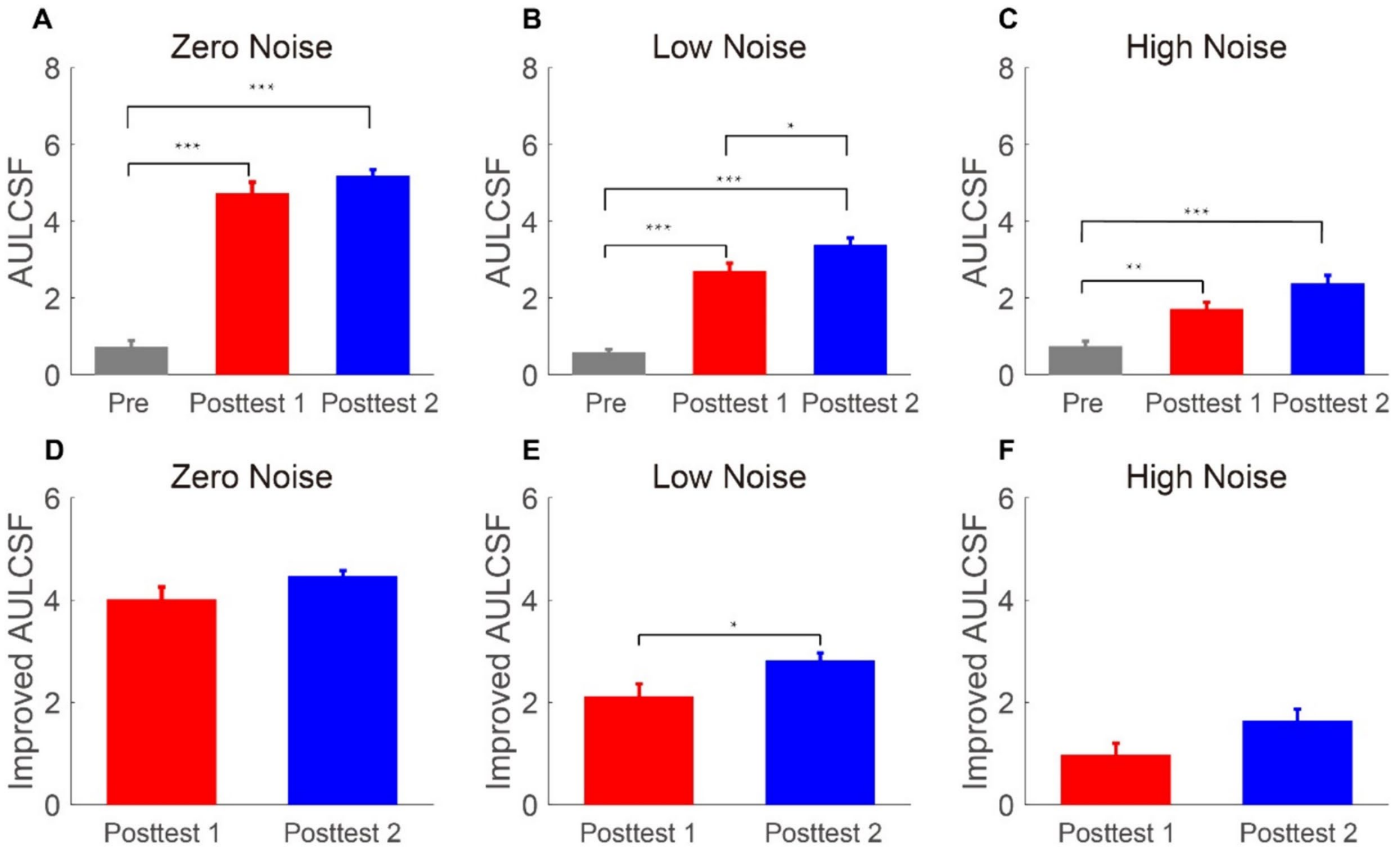
**(A–C)** Areas under log CS functions (log10 units) in the zero-, low-, and high-noise conditions when time points are pretest (gray bars), posttest 1 (blue bars), and posttest 2 (blue bars), respectively. **(D–F)** Areas under log-improved CS functions (log10 units) under zero-, low-, and high-noise levels when time points are posttest 1 (red bars) and posttest 2 (blue bars), respectively. The data collected from each subject were averaged. Standard errors (SEs) were represented by error bars.

To assess the impact of LASIK across SFs, the area under the log CSF (AULCSF) was shown in Figures 3A–C. To analyze the AULCSF, a repeated-measures ANOVA was employed, considering time points and external noise as within-subject factors. Significant findings were observed for the main effects of time point and external noise, as well as their interactive effect [*F*(2, 12) = 129.51, *p* < 0.001, $\eta_{p}^{2}$ = 0.956; *F*(2, 12) = 121.154, *p* < 0.001, $\eta_{p}^{2}$ = 0.953; *F*(4, 24) = 49.29, *p* < 0.001, $\eta_{p}^{2}$ = 0.891, respectively]. A simple-effect analysis indicated that (1) when noise was zero and high, the AULCSF at posttest 1 and posttest 2 was larger than that at pretest (all *p* < 0.004). However, there was no difference significant between posttest 1 and posttest 2 (all *p* > 0.098); (2) when noise was low, the AULCSF at posttest 2 was the best, followed by AULCSF at posttest 1 and AULCSF at pretest was the worst (all *p* < 0.018). Then, we determined the AULCSF increase at posttest 1 (the AULCSF at posttest 1 minus that at the pretest) and posttest 2 (the AULCSF at posttest 2 minus that at the pretest). A repeated-measures ANOVA was used on the AULCSF improvements with time point (posttest 1 and posttest 2) and external noise (zero, low, and high) as two within-subject variables (as shown in Figures 3D–F). The main effect of external noise was significant [*F*(2, 12) = 116.462, *p* < 0.001, $\eta_{p}^{2}$ = 0.951]; however, the main effect of the time point and the interaction between them were not significant [*F*(1, 6) = 5.757, *p* = 0.053, $\eta_{p}^{2}$ = 0.490; *F*(2, 12) = 0.506, *p* = 0.615, $\eta_{p}^{2}$ = 0.078, respectively]. The LSD test revealed that in the zero and high noise conditions, the AULCSF improvements had no difference between posttest 1 and posttest 2 (all *p* > 0.098); in the low noise condition, the AULCSF improvements at posttest 2 were significantly better than that at posttest 1 (*p* = 0.018). These findings suggested that the AULCSF recovers well after FS-LASIK and external noise modulates the recovery time.

**Figure 4:**
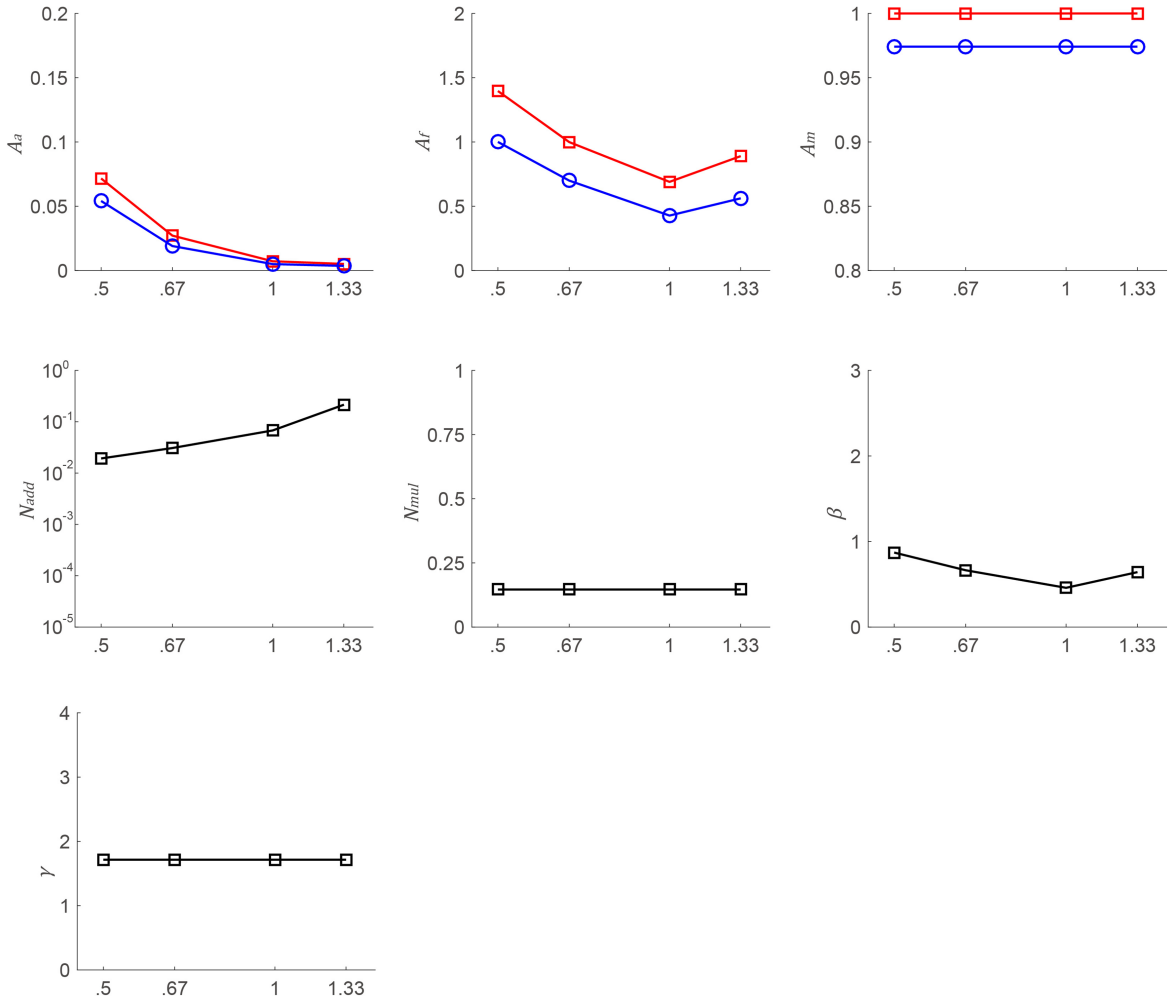
*A*_a_, *A*_f_, *A*_m_, *N*_add_, *N*_mul_, *β*, and *γ* as a function of SFs from the best-fitting model. The red line with squares and the blue line with circles denote data from posttest 1 and posttest 2, respectively.

To illustrate the mechanisms of the postoperative CS increase, the data were aggregated across participants and subsequently utilized to fit the PTM model (see Figure 4). We could make the assumption that *A*_m_ remains constant when the slopes of psychometric functions do not vary across different time points. To check this hypothesis, a repeated-measures ANOVA was conducted on the slopes, taking into account different time points (pretest, posttest 1, and posttest 2). The analysis revealed significant changes in the slopes over time, contrary to the initial assumption [*F*(2, 12) = 5.758, *p* = 0.018]. Thus, we put *A*_m_ into Equation 3. To identify the most appropriate model, two criteria need to be met: achieving a comparable *r*^2^ score to that of the full model and incorporating the fewest number of independent parameters. From the full to most reduced model, the *r*^2^ scores were 91.83% (*M*_0_, *A*_a_, *A*_m_, and *A*_f_ change), 61.61% (*M*_1_, *A*_a_, and *A*_f_ change), 25% (*M*_2_, *A*_m_, and *A*_f_ change), 63.18% (*M*_3_, *A*_a_, and *A*_m_ change), 74.88% (*M*_4_, *A*_a_ change), 20.79% (*M*_5_, *A*_m_ change), 20.17% (*M*_6_, *A*_f_ change), and 15.42% (*M*_7_, no change). To determine the best-fitting model, we conducted an *F*-test and found that the *r*^2^ of *M*_0_ was significantly better than that of others [*F*(2, 187) = 74.395, *p* < 0.001; *F*(9, 187) = 19.406, *p* < 0.001; *F*(8, 187) = 18.968, *p* < 0.001; *F*(10, 187) = 13.295, *p* < 0.001; *F*(10, 187) = 17.684, *p* < 0.001; *F*(16, 187) = 11.379, *p* < 0.001; *F*(18, 187) = 10.289, *p* < 0.001, respectively]. Thus, we choose the full model. To investigate the relationship among *A*_a_, *A*_f_, and SF, Pearson correlation analyses were conducted. Although there were no significant correlations between the above factors at posttest 1 and posttest 2 (all *p* > 0.084), the trends were observed from Figure 4, in which, *A*_a_ and *A*_f_ were decreased as SF rose.

### The relationship among SE, VA, and CS

To determine the relationship among SE, VA, and CS, we used the correlation coefficient analysis. VA was assessed using the logMAR vision chart, where a lower logMAR value corresponded to better VA. CS was determined using the AULCSF when the external noise was absent. SE has no significant correlation with VA and the AULCSF at the pretest, posttest 1, and posttest 2 (all *p* > 0.117). We also analyzed the correlation among improvements in SE, VA, and CS at posttest 1 (values at posttest 1 minus that at the pretest) and posttest 2 (values at posttest 2 minus that at the pretest); however, there were no significant correlations among them (all *p* > 0.215).

## Discussion

In this study, FS-LASIK has demonstrated both effectiveness and safety in correcting myopia, as evidenced by improvements in VA, SE, and CS following the surgery. We also found that changes in CS were influenced by external noise and SF. Moreover, we determined the underlying mechanisms of CS change by using the PTM model. The findings suggest that FS-LASIK reduced internal additive noise and enhanced the perceptual template, with both of these changes being dependent on SF.

Previous studies have confirmed that FS-LASIK enhances VA and SE. However, there remains uncertainty surrounding the influence of FS-LASIK on CS. This uncertainty can be attributed to two primary factors. First, some studies have relied on traditional methods, collecting CS over a limited range of SF (typically around 4 SFs) within a long duration. Second, alternative studies have applied a novel measurement method called the qCSF, based on the Bayesian algorithm (Lesmes, 2010). The qCSF offers the advantage of efficiently assessing CS across a broader range of SFs, encompassing 10 SFs, within a short duration. This efficiency helps mitigate the influence of fatigue. It is worth noting that previous researchers have employed the qCSF; however, their primary focus centered on the temporal aspect, specifically comparing CS before and after surgery, often neglecting the SF dimension. This approach has resulted in ambiguity regarding the specific changes in CS at individual SFs. To avoid the above shortages, we have addressed them by collecting CS data across 10 SFs using the qCSF. Our findings revealed that the effect of FS-LASIK on CS depended on time and SF. CS demonstrated improvements across a range of SFs (1.33–8 cpd), with the most significant enhancement occurring at mediate SFs. Furthermore, we observed a time-dependent increase in CS. For example, CS on postoperative day 7 exhibited notable improvements compared to that on postoperative day 1.

Another innovation of our study is the inclusion of external noise to simulate the real world. In order to investigate the operational impact under varying noise levels, we calculated the AULCSF, which provides a comprehensive assessment of CS across 10 SFs. We found consistent improvements in AULCSF across all noise levels when comparing pre-surgery and post-surgery data. These results indicate the efficacy of FS-LASIK in improving CS. Furthermore, external noise modulated the effect of time points on the AULCSF. Specifically, in the zero and high noise conditions, AULCSF was comparable between postoperative day 1 and postoperative day 7. However, in the low noise condition, AULCSF on postoperative day 7 was better than that on postoperative day 1. A similar trend emerged when assessing the improvements in AULCSF. Notably, in line with prior research, CS stabilizes at zero noise level by postoperative day 7 (Liu et al., 2016). Essentially, the recovery time following FS-LASIK spans approximately 1 week, shorter than that of other refractive surgeries such as SMILE and PRK. For example, Chiche et al. (2018) observed better postoperative CS in the FS-LASIK group compared to the SMILE group at 1 day and 1 week, although not at 1 month. Gao et al. (2022) demonstrated that CS recovery required 1 month following PRK.

The study examined changes in visual perception using the PTM, specifically, CS improvements were attributed to the reduction of internal noise and the enhancement of the perceptual template. The reduction of internal additive noise was accomplished through stimulus enhancement. The enhancement of the perceptual template, which refers to the improved ability to exclude external noise, results in improved performance by directing perceptual analysis toward the appropriate temporal duration, spatial region, and/or content characteristics of the signal stimulus (Bejjanki et al., 2014). Additionally, we expected to observe an increase in internal additive noise and perception template increase with rising SF. However, while a discernible trend is apparent, the correlation analysis is not significant. This may be due to the limited number of data, particularly the exclusion of data points above 1.33 cpd. This exclusion was due to poor preoperative CS and the emergence of the floor effect, resulting in an underestimation of CS within medium-high SF. In other words, our results suggest that CS shows the most improvements at medium SF, but it is plausible that the greatest enhancement actually occurs at high SF.

The current study not only investigates the surgical impact on various visual indicators but also assesses the relationship among these parameters. In line with prior research, FS-LASIK has shown effective improvements in vision function (i.e., VA, SE, and CS). It is worth noting that overcorrection was observed in two participants on postoperative day 1, but SE returned to normal by postoperative day 7, consistent with findings reported by Wei et al. (2020). For the majority of participants, SE on postoperative day 7 showed a slight decrease compared to postoperative day 1. These findings underscore the temporal requirement for the stabilization of surgical outcomes.

Although limited by the relatively short follow-up time, the study mainly focused on the early recovery of vision after FS-LASIK. Previous studies have shown that VA, SE, and CS stabilize within 1 week after FS-LASIK when comparing preoperative and 1 week postoperative data or data from longer postoperative periods (Kamiya et al., 2012; Tabacaru and Stanca, 2017; Gao et al., 2022). However, few studies have assessed vision during the early postoperative period, specifically changes in CS at 1 day and 7 days postoperatively. According to the study, VA and SE were comparable between postoperative day 1 and day 7, while CS improved until postoperative day 7.

In addition, no significant relationships were found among VA, SE, and CS at three time points (preoperative, postoperative day 1, and postoperative day 7), which might be attributed to the constraints imposed by the following factors: (1) the limited sample size; the number of participants complied with the experimental requirements, but significant trends are more likely to be observed in large sample size. (2) The dark lighting condition, wherein CS was measured in the dark environment. Following the findings of Gao et al. (2022), it was observed that in predicting photopic AULCSF, both SE and VA were significant predictors; however, in predicting mesopic AULCSF, neither SE nor VA emerged as significant predictors. (3) The limited measurement span; CS at each SF was assessed by detecting gratings with preset sizes. Preoperative CS might be overestimated when its value falls below the lowest limit of the measurement span, leading to an underestimated preoperative improvement in CS. In contrast, the minimum value of VA was accurately measured by shortening the distance between the subject and the chart. Consequently, the improvement in VA and CS appeared to be uncorrelated. In addition, the study was limited by not considering inflammatory factors secretion. In the study of Zhang et al. (2021), inflammatory mediators, such as nerve growth factor and tumor necrosis factor-α, changed significantly following FS-LASIK. These changes are likely involved in corneal wound healing and could serve as potential indicators for assessing dry eye post-surgery (Lambiase et al., 2000; Pajic et al., 2016; Wang et al., 2018).

Taking into consideration the aforementioned observations, we have formulated a plan for improving future research. First, we propose extending the duration of follow-up periods to enable a more comprehensive evaluation of the stability of FS-LASIK. Second, we advocate for the inclusion of larger sample sizes and the incorporation of multiple lighting conditions in our investigations to facilitate clearer and more conclusive conclusions. Third, we plan to evaluate the effect of FS-LASIK using a more comprehensive set of indices, including behavioral (e.g., VA and CS), physiological (e.g., tear inflammatory mediators), and subjective (e.g., surgical satisfaction) measures. We will then explore the relationships among these measures.

In summary, FS-LASIK demonstrates significant potential for comprehensive vision correction, particularly in CS, which exhibits enhanced performance even in the presence of external noise and SF variations. Consequently, we recommend the inclusion of CS as one of the standards to evaluate the outcomes following corrective surgery.

## Data Availability

The raw data supporting the conclusions of this article will be made available by the authors, without undue reservation.
